# Pharmacokinetics of Monoclonal Antibodies in Pediatrics: Model-Based Investigation on Allometric Scaling Exponents

**DOI:** 10.3390/pharmaceutics18050579

**Published:** 2026-05-07

**Authors:** Elvis K. Danso, Yuan Xiong, Mahesh N. Samtani, Zhenhua Xu

**Affiliations:** 1 Department of Clinical Pharmacology and Pharmacometrics, Johnson & Johnson Innovative Medicine, Spring House, PA 19002, USA; edanso@its.jnj.com (E.K.D.);; 2 Department of Clinical Pharmacology and Pharmacometrics, Johnson & Johnson Innovative Medicine, Raritan, NJ 08869, USA

**Keywords:** allometric scaling, pediatric, monoclonal antibodies, simulation, modeling, study design

## Abstract

**Methods**: This study explored key study design factors that could impact the precision of pediatric pharmacokinetics (PK) estimation. A virtual pediatric population was constructed, incorporating diverse body weight distribution sourced from the U.S. Centers for Disease Control and Prevention (CDC) growth charts. These generated weights were aggregated based on age ranges (2–5, 6–11, 12–17, and 2–17 y.o.), and different sample sizes were randomly selected to simulate PK concentrations over an approximately 5 half-lives period for a hypothetical monoclonal antibody. Throughout the simulations, the “true” allometric scaling exponents for the apparent volume of distribution and apparent clearance were consistently assumed to be 1.0 and 0.75, respectively, consistent with physiological and pharmacological knowledge for monoclonal antibodies. The impact of various pediatric study design factors on the model estimates of allometric exponents was then investigated by assuming the generated PK data as observed, with unknown PK parameters and allometric scaling exponent values. The data were subsequently fitted with population PK models, and estimated parameters were compared to “true” values to assess precision. Precision in estimated allometric exponents served as a marker for evaluating how effectively data from various study designs can inform the pediatric PK estimation. **Results**: Generally, estimates of allometric scaling exponents were more accurate with a larger sample size, proper PK sampling scheme, inclusion of densely sampled adult data, and a broader range of age. **Conclusions**: Considering the limitations in designing most pediatric studies, these findings support recent regulatory recommendations that standard allometric exponents should be considered in pediatric PK analysis for monoclonal antibodies in general.

## 1. Introduction

The use of therapeutic monoclonal antibodies (mAbs) has greatly increased for both adult and pediatric populations [1,2,3]. Generally, mAbs have a more targeted therapeutic effect and the potential for better safety profiles than small molecule drugs [4,5]. Understanding mAbs pharmacokinetics (PK) in pediatric patients is a regulatory requirement in drug development. However, collecting sufficient data in the pediatric population to adequately characterize PK profiles in children, in particular those of young ages, is often challenging, and approved adult-treatment drugs are usually prescribed off-label for pediatric use [6,7]. About 62–85% of pediatric prescribed drugs are used off-label [8], and this is even higher for children hospitalized in neonatal and pediatric intensive care units [9]. Pediatric studies not only involve smaller sample sizes relative to that of adults, but also in many cases, they may not include the same clinical endpoints as adult studies [6]. The aforementioned reasons invariably prolong new treatment alternatives for children and extend the drug development process for pediatrics, with approvals lagging behind adult approvals by an average of nine years [10]. Modeling and simulation have been a valuable approach to improve and accelerate the developmental process of pediatric studies without having the need to subject children to dose escalation regimens.

Allometric scaling has been commonly used to extrapolate PK data from adult to children for appropriate dose selection [11,12,13,14,15]. It has previously been shown that PK in children is predictable for mAbs exhibiting linear dynamics based on adult data and allometry [16]. This predictability, however, is contingent upon two key assumptions. First, the pediatric population should generally exclude neonates and young infants, as immunoglobulin G (IgG) concentrations undergo rapid, nonlinear changes during early development [17]. Additionally, the ontogeny of the neonatal Fc receptor (FcRn), which plays a role in mAb recycling, is not well-characterized and may not correlate directly with body weight [18]. Second, the monoclonal antibody should exhibit linear pharmacokinetics without significant target-mediated drug disposition. If the drug binds to a target with high affinity or exhibits nonlinear clearance, standard allometric scaling may be inadequate unless the target is fully saturated. These assumptions define the scope within which allometric extrapolation from adult to pediatric populations is considered reliable. The estimates and precision of the allometric exponents used in allometric scaling can be impacted and influenced by the limited sample size and how the PK data are collected and analyzed in pediatric studies. While the European Medicines Agency supports the use of fixed allometric exponents in the analysis of pediatric data because of data limitation [19], it is still estimated in model fitting. Although allometric scaling has been widely applied in pediatric pharmacokinetics, the novelty of our study lies in its systematic evaluation of how pediatric study design factors—such as sample size, age range, sampling scheme, and inclusion of adult data—influence the accuracy and bias of allometric exponent estimates. Previous studies, including Rowland et al. [20], have compiled typical allometric exponents for mAbs across species (e.g., 0.74 for clearance, 1.12 for V1, and 0.99 for V2), and Mahmood et al. [21] reported age-dependent exponents (e.g., ~0.9 for young children vs. 0.75 for adults). However, to our knowledge, no prior work has specifically examined the interplay of pediatric trial design parameters on the estimation of allometric scaling exponents. Our simulation-based approach quantifies how design constraints affect the reliability of exponent estimation. For instance, we show that small pediatric trials (e.g., *n* = 30 in a narrow age range) can yield highly variable exponent estimates (e.g., clearance exponent ranging from 0.41 to 1.09), whereas larger trials (*n* ≥ 60 with broader age ranges) produce estimates that are consistently close to the expected 0.75 value. These findings provide practical, quantitatively supported recommendations for optimizing pediatric trial design in the context of allometric scaling.

Our group has previously published two manuscripts focusing on pediatric PK predictions of mAbs. The simulations in the first study indicated that for therapeutic mAbs with linear PK, a weight threshold of 40 kg could generally be considered for pediatric subjects to receive the adult dosage [22]. In the subsequent study, the results affirmed that predicting PK in children aged 6–17 years is feasible for mAbs with linear PK before any pediatric data are available, based on adult data and weight effect using fixed allometric exponents of 1.0 for volume of distribution and 0.75 for clearance [16]. In this study, we assessed various study designs that could affect the estimation of allometric exponents in modeling when pediatric data become available. We first simulated an intensively sampled concentration–time profile of pediatric data using population PK parameters for a hypothetical mAb (see Section 2 for parameters and values) with allometric exponents of 1.0 and 0.75 for apparent volume of distribution (V/F) and apparent clearance (CL/F), respectively. Thereafter, we modeled various subsets of the simulated pediatric data to estimate the allometric exponents of V/F and CL/F under different scenarios, such as varying sample sizes, sampling schemes, and age groups. Additionally, pediatric data (sparsely sampled) were combined with adult data (intensively sampled) to mimic clinical trial scenarios to further explore how the allometric exponent could be impacted by combining pediatric and adult data.

Our study systematically evaluates the impact of various study design factors (including sample size, age/weight distribution, sampling schemes) on the characterization of pediatric PK for mAbs through PK simulations. This work aimed to investigate some key study design factors that could potentially affect the estimation of the allometric scaling exponents, highlighting the necessity of fixing them in pediatric studies with limited data, as suggested by the European Medicines Agency [19]. The accuracy of the estimation of the allometric scaling exponents should be data-driven, and therefore we explored the effects of sample size, sampling scheme, and age in relation to assumed ‘true’ simulated allometric exponents of 1.0 and 0.75, for V/F and CL/F, respectively. Furthermore, a hypothetical clinical trial scenario was modeled to elucidate the impact of adding Phase 1 adult data with intensive sampling to sparsely collected pediatric data on the estimation of allometric exponents of V/F and CL/F.

## 2. Materials and Methods

Given that this study was conducted entirely through simulations, it did not necessitate approval from an Institutional Review Board (IRB), nor was informed consent required.

### 2.1. Creation of Virtual Pediatric Weight Dataset

A virtual pediatric weight population was constructed with body weight distribution data from the Centers for Disease Control and Prevention (CDC) weight-for-age charts. Using the provided ages in months in the growth charts, the dataset was segmented into four age groups (2–5, 6–11, 12–17, and 2–17 y.o.) (Supplementary Figure S1A). Children younger than 2 years of age (i.e., neonates and infants) were not included in the simulations. The rationale behind this choice was that the PK model used in this study was developed from data in children aged 2 years and older. Additionally, there is limited availability of PK data for mAb use in children under 2 years, and extrapolating to this age group would require additional assumptions to account for rapidly changing physiology, such as the maturation of IgG levels and FcRn expression. More detailed considerations are provided in the Section 4.

A standard normal distribution (mean = 0, sd = 1) was randomly generated with 100 datapoints, representing the Z score ($\frac{x-mean}{sd}$) (Supplementary Figure S1A(i)). We selected 100 datapoints per age in months as a balance between computational efficiency and the need to capture a representative distribution of body weights. This number was sufficient to approximate the 3rd to 97th percentile range of the CDC growth curves. Increasing the number of samples beyond 100 did not materially affect the resulting weight distributions. Because the LMS (Lambda-Mu-Sigma) method was used with CDC growth chart parameters, the generated virtual weights closely matched real-world percentiles by design, including median and 5th/95th percentile values at key ages. For each age in months, 100 datapoints for weight were calculated using each of the 100 randomly normally distributed datapoints (Z), the corresponding values of the power of the Box–Cox transformation (L), median (M), and generalized coefficient of variation (S) for the specific age in months (Supplementary Figure S1A(ii)) using equation:

weight=M(1+ZLS)1L.

The generated weights for all ages in months (Supplementary Figure S1A(ii)) that corresponded to an age group were pooled together (Supplementary Figure S1A(iii)) and sampled randomly assuming different sample sizes of 30, 60, 120, 200, and 240 (Supplementary Figure S1A(iv)). This entire process from the generation of the standard normal distribution data (Supplementary Figure S1A(i)) through to the random selection of the sample sizes (Supplementary Figure S1A(iv)) was repeated each time for a new sample size.

### 2.2. Simulation of Dataset

Combining each age group’s sampled weights obtained from the CDC growth chart with a single SC dose of 2 mg/kg and population PK parameters from a hypothetical mAb study, simulations (repeated 100 times) of PK concentrations up to 56 days (approximately 5 half-lives) were conducted using the RxODE package (Version 0.9.1-0) in R (Version 3.4.1) (Supplementary Figure S1B,D). The population PK parameters of this mAb used for simulating a large PK dataset with intensive sampling scheme can be found in Table 1. They originated from an established PK model, developed internally from data collected in a Phase 3 study of a mAb representing a typical PK profile considered as representative of many known mAbs. This study included 173 pediatric participants aged 2 to 17 years.

In the simulations, V/F, CL/F, and Ka were given as follows:

(1) $$V/F=TV\times \exp\left(eta.V\right)\times (WT/70)$$

(2) $$CL/F=TCL\times \exp\left(eta.CL\right)\times {\left(WT/70\right)}^{0.75}$$

(3) $$Ka=TKa\times \exp\left(eta.Ka\right)$$

TV, TCL, and TKa are typical values of V/F, CL/F, and Ka, respectively. eta.V, eta.CL, and eta.Ka are the interindividual variabilities of V/F, CL/F, and Ka. WT represents weight, and for V/F and CL/F, it was normalized with an average adult body weight of 70 kg. Allometric exponents of V/F and CL/F were 1.0 and 0.75, respectively.

### 2.3. Virtual Adult Population

A virtual adult population (*n* = 200) was generated with a mean value of 70 kg and standard deviation of 10, and a lower and upper values of 60 kg and 110 kg, respectively. When adult data were needed for pooling with pediatric data for further analysis, 20 adult subjects were randomly sampled from this virtual adult population, which aligns with the typical design of a Phase 1 trial conducted with healthy male volunteers. We generated 200 random samples of adult weights to create a sufficiently large pool from which smaller subsets (20 adults) could be further sampled for different simulation scenarios. This approach introduces an additional level of variability across simulated datasets, mimicking scenarios in real-life clinical trials, and avoids overfitting to a fixed adult dataset. The adult weight distribution (mean 70 kg, SD 10 kg, range 60–110 kg) was selected to reflect a typical healthy adult male population and aligns with the standard 70 kg reference weight commonly used in PK allometric scaling. We did not explicitly model sex-based differences in weight; all virtual adults were treated generically. This simplification is consistent with the study’s focus on body weight as the primary covariate and not on sex-specific physiological differences.

### 2.4. Population PK Modeling

The first-order conditional estimation with interaction (FOCEI) method was used. To investigate the impact of sample sizes, sampling schemes, and age on model estimates of allometric exponents, PK concentration data following different scenarios of the above design factors were simulated and assumed as observed data from clinical trials. These hypothetical “observations” were subsequently fitted to population PK models, with the model PK parameters and allometric scaling exponents assumed unknown and to be estimated. Furthermore, a clinical hypothetical scenario was tested involving pooling intensively sampled Phase 1 data in adults with sparsely sampled Phase 3 pediatric data, and the results were compared with the estimates when only sparsely sampled pediatric data were used in modeling.

Data were extracted from the simulated dataset based on different sampling schemes to construct the NONMEM (Version 7.4.3, ICON plc, Hanover, MD, USA) modeling dataset, where a 0.6-day sampling window was applied around each planned sampling timepoint. The corresponding plasma concentrations were simulated at the randomly generated time points with this sampling window with intra-subject variability taken into consideration. The simulated concentration data were combined with weights and dosing information and next constructed for model fitting using NONMEM. For the allometric exponents of V/F and CL/F, sampling schemes (Supplemental Figure S1D) were:

(a) 3 samples (one at peak and two at terminal phase—days 4, 14, and 28),
(b) 5 samples (two before peak, one at peak, two at the terminal phase—days 1, 2, 4, 14 and 28),
(c) 8 samples (two before peak, one at peak, 5 after peak—days 1, 2, 4, 7, 14, 28, 42, and 56).

To mimic a hypothetical clinical scenario where the adult Phase 1 trial is sampled intensively and subsequent pediatric Phase 3 trial is sparsely sampled, adult data were combined with the pediatric data with the sampling scheme (Supplemental Figure S1D) as follows:

4, 14, 28 days (pediatric data only) vs. 4, 14, 28 days + 1, 2, 4, 7, 14, 28, 42, 56 days (pediatric + adult data).

## 3. Results

Section 3 is organized based on the study design factors (sample size, sampling scheme, and age). In each analysis, two of these design factors were held constant while the third factor was systematically varied to assess its impact. A graphical assessment of goodness-of-fit was employed to interpret the results. The age groups (2–5, 6–11, 12–17, and 2–17 y.o.) were kept separate for the analyses and were not pooled together.

### 3.1. Sample Size

**Figure 1:**
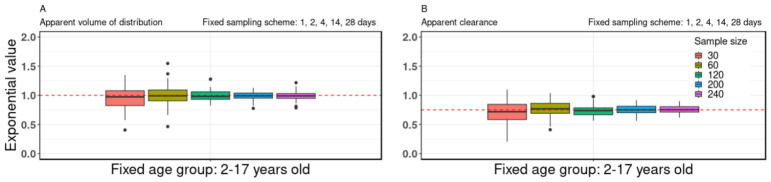
The impact of sample size on allometric scaling exponent. Box-plot data of allometric exponent values of (**A**) apparent volume of distribution and (**B**) apparent clearance for virtual pediatric population at a fixed age group of 2–17 y.o. and fixed sampling scheme on days 1, 2, 4, 14, and 28, with different sample sizes of 30, 60, 120, 200 and 240. In the box plots, the central line represents the median, the box denotes the interquartile range (25th–75th percentiles), whiskers extend to the most extreme non-outlier values (within 1.5× interquartile range), and individual points indicate outliers. Red dotted lines indicate the “true” value of the allometric exponent of the apparent volume of distribution and apparent clearance of 1.0 and 0.75, respectively. The accuracy of estimates of the allometric scaling exponents of both the apparent volume of distribution and apparent clearance increased with larger sample size.

The study explored the impact of varying sample sizes (30, 60, 120, 200, and 240) on the allometric scaling exponent while maintaining a consistent sampling scheme of 1, 2, 4, 14, and 28 days, as well as a fixed age group spanning from 2 to 17 years old. The findings indicated that as the sample size increased, there was a noticeable improvement in the accuracy of estimates for the allometric scaling exponents related to both the V/F and CL/F (see Figure 1). This trend remained consistent across various sampling schemes and age groups, as illustrated in Supplementary Figures S2 and S3, with additional details available in Supplementary Tables S1 and S2.

With a proper sample size of at least 60, the median values of the allometric exponents of both the apparent V/F and CL/F approximated the “true” allometric values as depicted in Figure 1. It is important to note that the threshold of 60 subjects is specific to the simulation scenario used in this study, which involved a hypothetical mAb with moderate inter-individual variability (~50% coefficient of variation) and a pediatric population aged 2–17 years. Inter-individual variability (IIV) was prespecified in the simulation framework based on values commonly reported in published adult and pediatric population PK analyses of IgG monoclonal antibodies, rather than estimated from a single dataset. An assumed IIV of ~50% for clearance and volume is consistent with prior experience. In practice, pediatric datasets are often insufficient to independently estimate IIV with the same precision as in adults; therefore, similar variability is commonly assumed once body size is accounted for. This assumption is supported by clinical and modeling evidence indicating comparable IIV between adults and children ≥2 years of age, consistent with the conserved nature of FcRn-mediated IgG clearance [1]. While we observed that bias in the estimated allometric exponents decreased markedly once the sample size reached approximately 60, this value should not be interpreted as a universal rule. The required sample size for reliable exponent estimation may vary depending on the PK properties of the drug, the variability in the population, and the age distribution of the study cohort. In scenarios with higher variability or more complex PK behavior, larger sample sizes may be necessary, whereas in lower-variability settings, fewer subjects might suffice. Therefore, our “≥60” recommendation should be viewed as illustrative of our specific modeling conditions rather than a generalizable threshold. Further details can be found in Supplementary Tables S1 and S2. Additionally, a wider body weight distribution from the broader age range (2–17 y.o.) was associated with less bias of estimates compared to the other age groups with narrower body weight distribution (2–5, 6–11, 12–17 y.o.), as illustrated in Supplementary Figures S2 and S3. For example, when the sample size was set to 30 and the sampling scheme involved days 1, 2, 4, 14 and 28, the inter-quartile ranges of the estimated allometric exponents for the broader age group (2–17 y.o.) were as precise as 0.82–1.08 and 0.58–0.85 for the V/F and CL/F, respectively (see Supplementary Tables S1 and S2; Ped: 1, 2, 4, 14, 28, sample size = 30). In contrast, the inter-quartile ranges of the estimated allometric exponents for the narrower age range of 2–5 y.o. could be as variable as 0.66–1.34 and 0.41–1.09, for the V/F and CL/F, respectively (see Supplementary Tables S1 and S2; Ped: 4, 14, 28, sample size = 30).

### 3.2. Sampling Scheme

**Figure 2:**
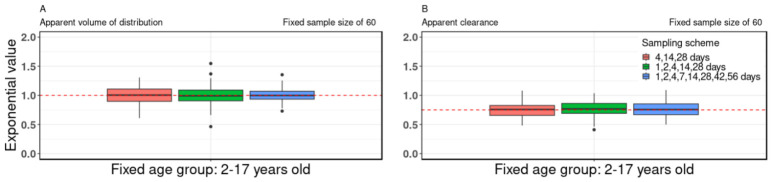
The impact of PK sampling scheme on the allometric scaling exponent. Box-plot data of the allometric exponent values of (**A**) apparent volume of distribution and (**B**) apparent clearance for virtual pediatric population at a fixed age group of 2–17 y.o. and fixed sample size of 60, with different sampling schemes of 4, 14, 28 days (red), 1, 2, 4, 14, 28 days (green), and 1, 2, 4, 7, 14, 28, 42, 56 days (blue). In the box plots, the central line represents the median, the box denotes the interquartile range (25th–75th percentiles), whiskers extend to the most extreme non-outlier values (within 1.5× interquartile range), and individual points indicate outliers. Red dotted lines indicate the “true” value of the allometric exponent of the apparent volume of distribution and apparent clearance of 1.0 and 0.75, respectively. The accuracy of estimates of the allometric scaling exponents of both the apparent volume of distribution and apparent clearance was similar across the different sampling schemes.

The study assessed the impact of sampling schemes, keeping a constant sample size of 60 and a fixed age group of 2–17 y.o. Notably, the results unveiled consistent estimates for the allometric scaling exponents of both the V/F and CL/F, as depicted in Figure 2. At a consistent sample size of 60 within the age group of 2–17 y.o., the inter-quartile ranges of the estimated allometric scaling exponents for the V/F were as follows for different sampling schemes: 0.90–1.11 (4, 14, 28 days), 0.91–1.09 (1, 2, 4, 14, 28 days), and 0.94–1.07 (1, 2, 4, 7, 14, 28, 42, 56 days) (see Supplementary Table S1). Additionally, at the fixed sample size of 60 within the age group of 2–17 y.o., the inter-quartile ranges of the estimated allometric scaling exponents for CL/F were as follows for specific sampling schemes: 0.66–0.83 (4, 14, 28 days), 0.69–0.86 (1, 2, 4, 14, 28 days), and 0.67–0.85 (1, 2, 4, 7, 14, 28, 42, 56 days) (refer to Supplementary Table S2). Consistency in these results was observed across various sample sizes and age groups, as depicted in Supplementary Figures S2 and S3 and detailed in Supplementary Tables S1 and S2. For instance, maintaining a fixed sample size of 240 for the age group of 6–11 y.o., the inter-quartile ranges of the estimated allometric scaling exponents for the V/F showed stability across different sampling schemes, measuring 0.89–1.10 for 4, 14, and 28 days, 0.90–1.07 for 1, 2, 4, 14, 28 days, and 0.92–1.09 for 1, 2, 4, 7, 14, 28, 42, 56 days (see Supplementary Table S1). Similarly, for CL/F at a fixed sample size of 240, the inter-quartile ranges demonstrated consistent values for the same sampling schemes, with measurements of 0.63–0.81 for 4, 14, 28 days, 0.66–0.83 for 1, 2, 4, 14, 28 days, and 0.67–0.85 for 1, 2, 4, 7, 14, 28, 42, 56 days (refer to Supplementary Table S2).

### 3.3. Age Range

**Figure 3:**
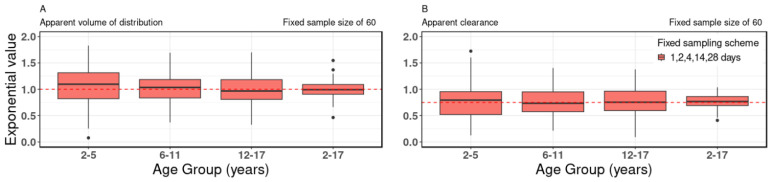
The impact of age range on allometric scaling exponent. Box-plot data of the allometric exponent values of (**A**) apparent volume of distribution and (**B**) apparent clearance for virtual pediatric population at a fixed sample size of 60 and fixed sampling scheme of 1, 2, 4, 14, 28 days, with different age groups of 2–5, 6–11, 12–17, and 2–17 y.o. In the box plots, the central line represents the median, the box denotes the interquartile range (25th–75th percentiles), whiskers extend to the most extreme non-outlier values (within 1.5× interquartile range), and individual points indicate outliers. Red dotted lines indicate the “true” value of the allometric exponent of apparent volume of distribution and apparent clearance of 1.0 and 0.75, respectively. The accuracy of the estimates of the allometric scaling exponents of both the apparent volume of distribution and apparent clearance increased for the age group of 2–17 y.o. when compared to the other age groups.

The study investigated the influence of age ranges on the allometric scaling exponent, maintaining a consistent sample size of 60 and a fixed sampling scheme of 1, 2, 4, 14, 28 days. The findings revealed that the estimates of the allometric scaling exponents for both the V/F and CL/F were less biased notably for the broader age group of 2–17 y.o., as depicted in Figure 3. This observed trend remained consistent across various sample sizes and age groups, as illustrated in Supplementary Figures S2 and S3, with additional detailed information available in Supplementary Tables S1 and S2. As an illustration, at a fixed sample size of 60 with a sampling scheme of 1, 2, 4, 14, and 28 days, the inter-quartile ranges of the estimated allometric exponent for the V/F were as follows: 0.82–1.31 for 2–5 y.o., 0.83–1.19 for 6–11 y.o., 0.81–1.18 for 12–17 y.o., and 0.91–1.09 for the broader age range of 2–17 y.o. (refer to Supplementary Table S1). Similarly, for CL/F, at the same fixed sample size and sampling scheme, the inter-quartile ranges were 0.52–0.96 for 2–5 y.o., 0.57–0.95 for 6–11 y.o., 0.59–0.96 for 12–17 y.o., and 0.69–0.86 for the overall age group of 2–17 y.o. (see Supplementary Table S2).

### 3.4. Combining Adult Data

**Figure 4:**
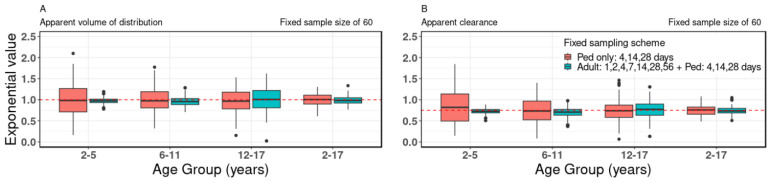
Impact of intensively sampled adult data when combined with sparsely sampled pediatric data. Box-plot data of allometric exponent values of (**A**) apparent volume of distribution and (**B**) apparent clearance for virtual pediatric population at a fixed sample size of 60 and fixed sampling scheme of 4, 14, 28 days (for pediatric data only) and 1, 2, 4, 7, 14, 28, 56 days + 4, 14, 28 days (for the adult and pediatric data, respectively, combined), with different age groups of 2–5, 6–11, 12–17, and 2–17 y.o. In the box plots, the central line represents the median, the box denotes the interquartile range (25th–75th percentiles), whiskers extend to the most extreme non-outlier values (within 1.5× interquartile range), and individual points indicate outliers. Red dotted lines indicate the “true” value of the allometric exponent of apparent volume of distribution and apparent clearance of 1.0 and 0.75, respectively. The accuracy of estimates of the allometric scaling exponents of both the apparent volume of distribution and apparent clearance increased for all age groups when the pediatric only data were compared to the data comprising both the pediatric and adult data, except for the adolescent age group of 12–17 y.o. The combination of the intensively sampled adult data and sparsely sample pediatric data mimic the clinical trial scenario for adult Phase 1 and pediatric Phase 3 trials, respectively.

The study delved into the impact of combining sparsely sampled pediatric data with intensively sampled adult data, a hypothetical scenario for pediatric PK modeling. Focusing on a fixed sample size of 60 and fixed sparsely sampled schemes for pediatric data (4, 14, 28 days), and when combined with intensively sampled adult data (1, 2, 4, 7, 14, 28, 56 days), the estimates for both V/F and CL/F varied across all age groups, with the exception of the adolescent age group (12–17 y.o.) where consistency was maintained (refer to Figure 4, Figures S4 and S5. This consistent trend observed in the adolescent age group was also evident across various sample sizes and different sampling schemes.

For V/F, comparing the inter-quartile ranges of allometric scaling exponents for the combined group (adult + pediatric) to pediatric-only, at a sample size of 60, yielded the following ranges: 0.93–1.01 vs. 0.71–1.26 (2–5 y.o.), 0.88–1.03 vs. 0.81–1.19 (6–11 y.o.), 0.81–1.22 vs. 0.78–1.18 (12–17 y.o.) and 0.92–1.05 vs. 0.90–1.11 (2–17 y.o.) (see Supplementary Table S3 and Supplementary Figure S4). Similarly, for CL/F, the inter-quartile ranges for the combined group (adult + pediatric) compared to pediatric-only were 0.69–0.77 vs. 0.49–1.14 (2–5 y.o.), 0.63–0.77 vs. 0.52–0.97 (6–11 y.o.), 0.63–0.90 vs. 0.58–0.87 (12–17 y.o.), and 0.69–0.80 vs. 0.66–0.83 (2–17 y.o.) (see Supplementary Table S4 and Supplementary Figure S5).

### 3.5. Body Weight Distribution

**Figure 5:**
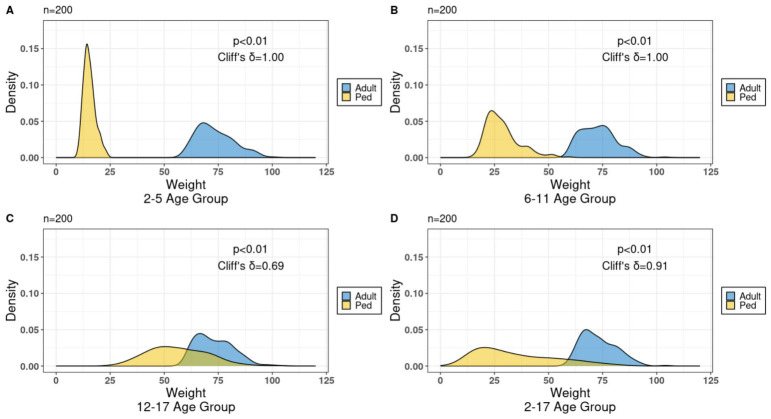
Body weight distribution of the adult and pediatric data (*n* = 200) for the various age groups (**A**) 2–5 y.o.; (**B**) 6–11 y.o.; (**C**) 12–17 y.o.; and (**D**) 2–17 y.o.

The body weight distribution between adult and pediatric data was distinct for various age groups. Specifically, for age groups 2–5 years old (refer to Figure 5A) and 6–11 years old (Figure 5B), as well as the broader range of 2–17 years old (Figure 5D), clear distinctions were observed. Although there was a small overlap at the tails within the wider age group (Figure 5D), clear distinctions were observed between adult and pediatric body weight distributions. In these groups, statistical comparison using a two-sample Kolmogorov–Smirnov (KS) test demonstrated significant distributional differences (*p* < 0.01), with Cliff’s delta (δ = 1.00 for 2–5 and 6–11 years; δ = 0.91 for 2–17 years) indicating near-complete separation between adult and pediatric weights, despite minimal tail overlap in the pooled 2–17 group.

Contrastingly, the adolescent age group (12–17 years old) exhibited a significant portion of body weights overlapping with adult weights (refer to Figure 5C). Although the KS test remained statistically significant (*p* < 0.01), the reduced effect size (Cliff’s δ = 0.69) reflects substantial overlap between adolescent and adult weight distributions, consistent with partial convergence toward adult body size in this age range.

## 4. Discussion

Model-based allometry that extrapolates data from adults to pediatrics based on body weight scaling has become a standard approach for pediatric drug development studies [23,24], as pediatric data are often limited in clinical studies. This is because limited pediatric data are usually generated for clinical studies due to ethical, regulations and operational challenges, and computational modeling [25,26,27,28], and simulations could be used to generate virtual pediatric data, and with allometry, help bridge the studies between adult and children.

Allometric scaling is a widely utilized method for predicting the PK of mAbs and other therapeutic proteins in pediatric patients by leveraging adult data. It relies on body weight or surface area as a scaling factor to account for differences in drug disposition between populations. Allometric scaling is a useful approach in pediatric drug development because it allows for safer and more effective dosing while minimizing the need for extensive pediatric clinical trials. Our analysis assumes that body weight is the primary covariate and does not explicitly account for developmental factors (e.g., maturation of organ function, FcRn levels) or disease-related effects on mAb pharmacokinetics. Therefore, the conclusions are most directly applicable to mAbs with linear PK in pediatric populations older than 2 years, where body size is the dominant determinant of PK. We acknowledge that for neonates and infants, immunoglobulin levels and possibly FcRn expression undergo rapid maturation that is not captured by body weight alone, and that for therapeutic proteins with target-mediated clearance, allometric scaling may be less predictive unless the target is fully saturated. These are important limitations of our study.

While our study is based on simulated data, we emphasize that the PK parameters used were derived from a real Phase 3 clinical study involving 173 pediatric patients. These values represent a typical mAb with linear PK and were selected to reflect clinically relevant properties. Nevertheless, it is possible that simulation-based studies cannot fully capture the variability and complexity of real-world pediatric populations. Factors such as disease state, adherence, assay variability, and comorbidities (e.g., juvenile idiopathic arthritis or pediatric Crohn’s disease) can significantly influence mAb PK. As such, our findings should be interpreted as qualitative guidance for optimizing pediatric trial design rather than prescriptive clinical recommendations. Future work should aim to validate these findings using real-world pediatric PK datasets. For example, comparing our simulation-based conclusions with observed data from therapies like adalimumab or infliximab could help assess the generalizability of our results. Our prior work [16] demonstrated the feasibility of adult-to-child PK extrapolation for mAbs using actual clinical data. These efforts support the plausibility of our assumptions in the context of linear mAbs, while highlighting the need for further empirical validation.

Our model intentionally assumes that body weight is the sole covariate influencing mAb PK and does not incorporate age-dependent physiological maturation (e.g., hepatic or renal function, FcRn ontogeny) or disease-state variability. These simplifications were made to isolate the effects of study design factors (e.g., sample size, age range, sampling scheme) on the estimation of allometric exponents under a best-case scenario. We acknowledge that in real-world pediatric populations, particularly in younger children (e.g., 2–5 years), ontogenic changes such as FcRn-mediated recycling and renal filtration maturity can significantly influence mAb disposition. For example, Mahmood et al. [21] reported higher allometric exponents (~0.9) for clearance in 2–5 year-olds compared to 0.75 in adults, highlighting the impact of developmental physiology. Additionally, our model does not account for disease-related alterations in PK, such as those observed in pediatric patients with chronic inflammatory conditions (e.g., juvenile idiopathic arthritis). Finally, we assumed linear PK and did not model target-mediated drug disposition (TMDD), which is common in many therapeutic mAbs and introduces nonlinearity in clearance. In such cases, weight-based allometric scaling may be insufficient unless the target is saturated. These factors should be considered when applying our findings to clinical scenarios, and future work should explore how these biological complexities interact with study design to influence parameter estimation.

While it is intuitive that larger sample sizes and broader age or weight ranges improve the precision of parameter estimates, our study provides quantitative benchmarks that go beyond this general expectation. Specifically, we demonstrate that precision gains begin to plateau beyond a sample size of approximately 60 subjects, offering a practical threshold for study planning. This insight helps researchers balance statistical rigor with feasibility in pediatric trial design. Moreover, our analysis reveals that the benefit of adding adult data is not uniform across all pediatric age groups. For younger children (e.g., 2–5 years), adult data significantly enhance the stability of exponent estimates, whereas for adolescents (12–17 years), who already share similar body weights with adults, the added value is minimal. These findings underscore the importance of tailoring data integration strategies to the specific age distribution of the pediatric cohort—an insight that is not immediately apparent without simulation-based evaluation. By quantifying these effects, our study contributes a practical framework for optimizing pediatric PK study designs. In our combined adult–pediatric simulations, we used a 3:1 ratio (60 pediatric to 20 adult subjects) to reflect a typical scenario where a pediatric Phase 3 trial is supplemented by a smaller Phase 1 adult study. However, we acknowledge that in real-world settings, adult datasets are often much larger due to prior clinical development. This imbalance poses a risk of adult data dominating the model fitting process, potentially skewing the estimation of allometric exponents toward adult physiology. This is particularly challenging when pediatric patients differ significantly from healthy adults in terms of disease state or developmental physiology.

While our simulations showed that adding adult data improved parameter estimation in most pediatric subgroups, the benefit was limited in adolescents due to overlapping weight distributions with adults. To mitigate the risk of adult-data dominance, we recommend that investigators consider strategies such as down-weighting adult data, applying hierarchical modeling frameworks, or conducting sensitivity analyses across varying adult-to-pediatric ratios. These approaches can help preserve the integrity of pediatric-specific parameter estimation. We also recognize that our study did not explore a range of adult–pediatric ratios, and we identify this as an important area for future research. Our findings also have implications for regulatory guidance on pediatric PK modeling. When pediatric studies are sufficiently powered (e.g., ≥60 subjects in our scenario) and include a broad age range, our simulations suggest that allometric exponents for clearance and volume can be estimated with acceptable precision and minimal bias. In such cases, fixing the exponents a priori may not be necessary, as the data can support reliable estimation. Conversely, our results reinforce current EMA guidance that recommends fixing exponents in data-limited pediatric analyses. In scenarios with small sample sizes or narrow age ranges, we observed that freely estimating exponents often led to biased or unstable values. Furthermore, building on the work of Mahmood et al. [21] our results support the idea that age-stratified exponent strategies may be appropriate. For example, in very young children (e.g., 2–5 years), a slightly higher exponent than 0.75 for clearance may better reflect physiological ontogeny, while the conventional 0.75 may remain suitable for older children. Although we do not propose formal revisions to existing regulatory policies, our findings provide quantitative support for a more nuanced application of current guidance and may inform future discussions on pediatric extrapolation strategies. Results obtained in this study demonstrated that the allometric exponents of both V/F and CL/F are significantly affected by study designs. The results revealed that the allometric exponents for both the V/F and CL/F were less biased and approached the “true” allometric exponents as the sample size increased. This trend held true across various age groups and sampling schemes, as illustrated in Supplementary Figures S2 and S3. Based on the results, it can be suggested that a sample size of less than 60 might increase the bias in the estimation of the allometric exponents. Pediatric studies are often conducted with smaller sample sizes due to ethical constraints and study challenges. Thus, following a standard approach to fix allometric exponents a priori could alleviate bias introduced by study design [19]. While it is true that many published allometric exponents for mAbs fall within a relatively narrow range (e.g., 0.75–0.90 for clearance), our results demonstrate that in scenarios with limited pediatric data, the allometric exponents can be substantially inaccurate. For example, in one simulation involving only 30 children aged 2–5 years, the interquartile range of the estimated clearance exponent spanned from 0.41 to 1.09. Such variability highlights the risk of severely mis-estimating the exponent when attempting to estimate it freely with insufficient data. Therefore, although small differences within the 0.75–0.90 range may have limited clinical impact due to population variability, the potential for much larger deviations in underpowered studies underscores the importance of fixing the exponent in such cases. This consideration was a key motivation for our study and supports the regulatory recommendation to fix allometric exponents when pediatric data are sparse. Furthermore, in the design of pediatric studies, it is crucial to consider the utilization of a wide range of age and body weights such as the 2–17 y.o. age group (encompassing a wider body weight group), which exhibited better results compared to the other age groups that were narrower.

The peak concentration of day 4 is a representative case for subcutaneous (SC) administration of mAbs. mAbs are typically administered via parenteral routes due to their large molecular size and relative polarity, which limits their permeability through the gastrointestinal mucosa [29]. While intravenous administration remains the most common method, SC administration offers greater convenience for patients and are therefore also widely used [30]. PK sampling that excludes either the early absorption and late terminal phases (i.e., days 1, 2, 42 and 56) (Supplementary Figure S2A) or the late terminal phase only (i.e., days 42 and 56) (Supplementary Figure S2B) did not influence the accuracy of the allometric exponents of the *V*/*F* when their respective age groups were compared to the full PK sampling scheme from days 1–56 (Supplementary Figure S2C). Similarly for *CL/F*, without the early absorption and late terminal phases (i.e., days 1, 2, 42 and 56) (Supplementary Figure S3A) or the late terminal phase only (i.e., days 42 and 56) (Supplementary Figure S3B), the accuracy of the allometric exponents did not change when compared to the full range PK sampling from days 1–56 (Supplementary Figure S3C). This indicates that including only the peak concentration data (day 4) and early to middle terminal phase data in the analyses is sufficient to characterize PK for a typical mAb using a 1-compartment model. For this exploratory analysis, the pre-peak samples (day 1 and day 2) were considered less informative compared to the peak sample around day 4. Similarly, late terminal phase samples (days 42 and 56) showed concentrations reduced to below 7% of the peak concentration, which likely adds minimal information if we already have samples from days 14 and 28. Additionally, it contributed to a reduction in the overall data requirement from pediatric populations. This insight is particularly valuable, especially given the often-limited data available in pediatric studies. In many pediatric trials, predominantly trough samples are collected, enabling sufficient characterization of CL/F. However, non-trough levels, particularly peak concentrations, can offer valuable insights for the adequate characterization of V/F and contribute to model stability.

The study revealed that the broader age and body weight range (2–17 y.o.) yielded less biased estimates of the allometric exponents when compared to the narrow range of body weight (Supplementary Figures S2 and S3). A broad age range in PK studies facilitates a more comprehensive understanding of how drugs behave in pediatric populations, aiding dose optimization, enhancing generalizability, and meeting regulatory requirements. Despite the challenges, the inclusion of older children first and then younger children, before moving on to vulnerable populations may be necessary. In pediatric PK studies this is crucial for a holistic and meaningful understanding of drug behavior in the entire pediatric population. This approach supports safer and more effective drug use in pediatric patients across different age ranges.

We further combined pediatric data with adult data to mimic a hypothetical scenario where sparsely sampled data from a Phase 3 pediatric study are combined with intensively sampled data from an adult study [10]. Our results showed that apart from the age group of 12–17 years old, the accuracy of the allometric exponents of V/F improved when we added the intensively sampled adult data (days 1, 2, 4, 7, 14, 28, 42, and 56) to the sparely sampled pediatric data (days 4, 14, and 28) (Supplementary Figure S4). This was also the case for the allometric exponents of CL/F (Supplemental Figure S5). This indicates that there is additional benefit of improving estimates when adult and pediatric data are combined such as model stability and convergence. However, there should be careful consideration when combining adult and pediatric data. If the pooled dataset has the majority of the information coming from adults, this can move the estimates toward those derived from the adult dataset, particularly in terms of parameter scaling, leading to inaccurate interpretations of the pediatric population’s characteristics. Thus, pooling pediatric data with adult data might not be ideal because adult samples typically outnumber pediatric samples by a large margin. It is also important to highlight that rich adult PK data usually come from Phase 1 studies in healthy subjects, whereas pediatric data often come from pediatric patients, which may make pooling the datasets difficult. This discrepancy can result in allometric scaling exponents that do not accurately reflect the pediatric patient population, potentially leading to incorrect conclusions based on healthy adult data. It is also noted that the combination of data from the adolescent age group (12–17 y.o.) and Phase 1 data from adults did not improve the estimation of the allometric exponents (Supplementary Figures S4 and S5). This could be attributed to the similarity in body weight distribution between adolescents (12–17 y.o.) and adults (Figure 5C), suggesting that body weight range could distort the estimation of the allometric exponents. Previous studies [16,18] suggest that a body weight threshold of 40 kg is generally recommended for pediatric patients to receive the same fixed adult dosage of mAbs with linear PK. While this threshold serves as a useful starting point, it is neither definitive nor rigid. Our study supports this claim for the adolescent age 12–17 years (average weight of 53 kg) as the data showed consistency between analyses focused solely on pediatric patients and those incorporating both pediatric and adult data (Supplementary Figures S4 and S5). All other age groups had average weights below the 40 kg threshold. Notably, the inclusion of adult data to the 12–17 age group (average weight of 53 kg) did not alter the exponents, indicating that the PK parameters for adolescents are comparable to those of adults. This finding suggests that adolescents may safely receive adult dosages, as their drug metabolism and response appear similar to those of adult patients. However, we recognize and acknowledge that our simulations are based on a narrowly defined scenario and should not serve as the sole reference for guiding pediatric dosing strategies. It is essential to recognize that including pediatric patients with extreme body weights (whether unusually low or high) can significantly distort the estimation of allometric exponents. These outliers can exert disproportionate influence, skewing results and potentially resulting in biased scaling parameters. Specifically, extremely low body weights may correlate with altered drug metabolism or clearance, while excessively high body weights can introduce complexities such as an increased volume of distribution. Addressing how these factors interact with the estimated scaling exponents is crucial, highlighting the importance of fixing the allometric exponent.

The European Medicines Agency suggests employing fixed allometric exponents for analyzing pediatric data [19]. Given that pediatric study sample sizes are typically small and often challenging, adopting standard values may offer a practical solution providing reasonably accurate PK projections. The rationale of this approach is also supported by the extensive experience accumulated from developing relevant biologic therapeutics in pediatrics [22]. In cases where a change in pediatrics PK is observed, they may be likely attributed more to disease-related factors rather than allometric exponents. By maintaining fixed allometric exponents, one can better determine PK differences relative to adults, potentially elucidating additional effects attributed to disease, population, or indication. A previous study outlines the sample size needed per age group for precisely estimating clearance based on the level of between-subject variability, aiming to achieve at least 80% power [31]. This methodology can also be applied to the volume of distribution.

Although body weight is typically considered one of the most influential covariates in the 2–17 y.o. age group, other age-dependent factors such as hepatic function, disease status and maturation-related processes [13,32,33] may also impact PK parameters and contribute to changes in drug disposition and elimination. Mahmood et al. [21] suggested an age-dependent exponent of 0.9 for children aged 2–5 years and 0.75 for adults, while other studies have proposed an exponent ranging between 0.75 and 0.9 in adults. However, in this study, we adopted a simplified approach, considering body weight as the only influential covariate and assuming that other factors remain constant or have minimal impact in PK parameters across age. This study focused exclusively on mAbs, and complexities like ontogeny that are relevant to small molecules were not included in this analysis. Children, including infants, can maintain immunoglobulin homeostasis, suggesting that they can effectively metabolize mAbs. As a result, significant age-related developmental differences are unlikely [1]. However, PK data for children under 2 years old remain scarce [1]. Consequently, this manuscript focused on PK simulations for children aged 2 to 17 years.

## 5. Conclusions

Given its feasibility and practicality, pediatric data with a proper sample size were shown to be adequate for pediatric allometric scaling, without considerable bias. Furthermore, except for the adolescent age group, combining sparsely sampled pediatric data with intensively sampled adult data may improve the accuracy of estimates. However, caution must be exercised due to potential influences arising from imbalanced sample sizes or differences in disease status. With the tested hypothetical mAb PK profile, sparse sampling of pediatric data was sufficient to determine pediatric allometric exponents for both the V/F and CL/F, with little bias. Therefore, careful consideration of study designs is essential when planning pediatric studies to accurately characterize PK in this population. When the sample size is limited due to practical reasons, following the recent recommendation by regulatory agencies to fix the allometric exponent would be a less-risky approach. Additionally, when interpreting and drawing conclusions from pediatric PK results, it is essential to do so while considering the intricacies of study designs and their associated data limitations.

This study has several limitations. First, our simulations were limited to children aged 2–17 years, as the PK model was derived from data in that age range. Therefore, extrapolation of our findings to infants and neonates (<2 years) is uncertain due to rapid developmental changes in that population. Second, we used a simplified model that considered only body weight as an influential covariate and assumed linear PK. We did not incorporate age-dependent physiological maturation (e.g., hepatic or renal function, FcRn ontogeny), disease-state variability, or nonlinear target-mediated drug disposition. These simplifications were intentional to isolate the effects of study design, but they limit the applicability of our findings to more complex real-world scenarios. Third, our conclusions are based on a single hypothetical mAb profile and simulated data. While this approach allowed us to focus on the evaluation of the effect of body weight, it does not capture real-world complexities such as inter-patient variability, adherence, or assay differences. As such, our quantitative recommendations (e.g., sample size thresholds) should be interpreted with caution. Future work should validate these findings using real pediatric PK data and explore mAb with diverse PK properties to assess the robustness and generalizability of our conclusions.

## Figures and Tables

**Table 1:** Population pharmacokinetic parameters and values of monoclonal antibodies used for simulating the dataset. These originated from an established PK model, developed internally from data collected in a Phase 3 study of a monoclonal antibody (mAb), representing a typical PK profile considered as representative of many known mAbs. This study included 173 pediatric participants aged 2 to 17 years.

Population PK Parameters	Values
Apparent volume of distribution (*V/F*)	18 L (interindividual variability 46%)
Apparent clearance (*CL/F*)	1 L/day (interindividual variability 49%)
Absorption rate constant (*Ka*)	0.9 day^−1^ (interindividual variability 50%)
Correlation between *V/F* and *CL/F*	0.7
Proportional residual variability	25%
Allometric exponent of *V/F*	1
Allometric exponent of *CL/F*	0.75

## Data Availability

Original datasets are available from the corresponding author on request.

## Supplementary Materials

The following supporting information can be downloaded at: https://www.mdpi.com/article/10.3390/pharmaceutics18050579/s1, Figure S1: Schematic of how pediatric data was generated, simulated, and modeled to determine allometric exponents. (A) Using data from CDC weight-for-age charts, one hundred weights were generated for each age (in month) record with a standard normal distribution (weight=M(1+ZLS)1L). M represents median; Z represents Z score values generated from the standard normal distribution with 100 datapoints; L is power in the Box-Cox transformation; S is generalized coefficient of variation. All the individually generated one hundred weights for each age bracket were pooled together from which samples were randomly selected for 30, 60, 120, 200 and 240 different body weights. (B) Using RxODE, the generated pediatric data were simulated with the parameters provided (C) Using NONMEM, simulated data obtained from different sampling schemes and design factors were modeled to estimate the allometric exponents. Figure S2: Box-plot data of allometric exponent values of apparent volume of distribution for the virtual pediatric population for different age groups (2–5, 6–11, 12–17 and 2–17 y.o) and sample sizes of 30, 60, 120, 200 and 240, sampled on days (A) 4, 14, and 28 (B) 1, 2, 4, 14, and 28, and (C) 1, 2, 4, 7, 14, 28, 42, and 56. In the box plots, the central line represents the median, the box denotes the interquartile range (25th–75th percentiles), whiskers extend to the most extreme non-outlier values (within 1.5× interquartile range), and individual points indicate outliers. Red dotted lines indicate “true” value of allometric exponent of apparent volume of distribution of 1.0. Figure S3: Box-plot data of allometric exponent values of apparent clearance for the virtual pediatric population for different age groups (2–5, 6–11, 12–17 and 2–17 y.o) and sample sizes of 30, 60, 120, 200 and 240, sampled on days (A) 4, 14, and 28 (B) 1, 2, 4, 14, and 28, and (C) 1, 2, 4, 7, 14, 28, 42, and 56. In the box plots, the central line represents the median, the box denotes the interquartile range (25th–75th percentiles), whiskers extend to the most extreme non-outlier values (within 1.5× interquartile range), and individual points indicate outliers. Red dotted lines indicate “true” value of allometric exponent of apparent clearance of 0.75. Figure S4: Combined intensively sampled adult data (Phase 1 trial) and sparsely sample pediatric data (Phase 3 trial) to mimic clinical trial scenario. Box-plot data of allometric exponent values of apparent volume of distribution for the virtual pediatric population for different age groups (2–5, 6–11, 12–17 and 2–17 y.o) and sample sizes of 30, 60, 120, 200 and 240, sampled on days (A) 4, 14, and 28 for the pediatric data and (B) 1, 2, 4, 7, 14, 28, 42, and 56 for adult data combined with 4, 14, and 28 for pediatric data. In the box plots, the central line represents the median, the box denotes the interquartile range (25th–75th percentiles), whiskers extend to the most extreme non-outlier values (within 1.5× interquartile range), and individual points indicate outliers. Red dotted lines indicate “true” value of allometric exponent of apparent volume of distribution of 1.0. Peak concentration is achieved around day 4. Figure S5: Combined intensively sampled adult data (Phase 1 trial) and sparsely sample pediatric data (Phase 3 trial) to mimic clinical trial scenario. Box-plot data of allometric exponent values of apparent clearance for the virtual pediatric population for different age groups (2–5, 6–11, 12–17 and 2–17 y.o) and sample sizes of 30, 60, 120, 200 and 240, sampled on days (A) 4, 14 and 28 for the pediatric data and (B) 1, 2, 4, 7, 14, 28, 42, and 56 for adult data combined with 4, 14 and 28 for pediatric data. In the box plots, the central line represents the median, the box denotes the interquartile range (25th–75th percentiles), whiskers extend to the most extreme non-outlier values (within 1.5× interquartile range), and individual points indicate outliers. Red dotted lines indicate “true” value of allometric exponent of apparent clearance of 0.75. Peak concentration is achieved around day 4. Table S1: Statistics of allometric exponent values of apparent volume of distribution for the virtual pediatric population for different age groups (2–5, 6–11, 12–17 and 2–17 y.o) and sample sizes of 30, 60, 120, 200 and 240, sampled on days (A) 4, 14, and 28 (B) 1, 2, 4, 14, and 28, and (C) 1, 2, 4, 7, 14, 28, 42, and 56. Table S2: Statistics of allometric exponent values of apparent clearance for the virtual pediatric population for different age groups (2–5, 6–11, 12–17 and 2–17 y.o) and sample sizes of 30, 60, 120, 200 and 240, sampled on days (A) 4, 14, and 28 (B) 1, 2, 4, 14, and 28, and (C) 1, 2, 4, 7, 14, 28, 42, and 56. Table S3: Statistics of allometric exponent values of apparent volume of distribution for the virtual pediatric population for different age groups (2–5, 6–11, 12–17 and 2–17 y.o) and sample sizes of 30, 60, 120, 200 and 240, sampled on days (A) 4, 14, and 28 for the pediatric data and (B) 1, 2, 4, 7, 14, 28, 42, and 56 for adult data combined with 4, 14, and 28 for pediatric data. Table S4: Statistics of allometric exponent values of apparent clearance for the virtual pediatric population for different age groups (2–5, 6–11, 12–17 and 2–17 y.o) and sample sizes of 30, 60, 120, 200 and 240, sampled on days (A) 4, 14 and 28 for the pediatric data and (B) 1, 2, 4, 7, 14, 28, 42, and 56 for adult data combined with 4, 14 and 28 for pediatric data.
