# Supplementary material for: Pharmacokinetics of Monoclonal Antibodies in Pediatrics: Model-Based Investigation on Allometric Scaling Exponents
Source: Pharmaceutics. 2026 May 7;18(5):579. doi: 10.3390/pharmaceutics18050579 (PMC13210262; doi:10.3390/pharmaceutics18050579)
Supplement: Supplementary file 1 [file pharmaceutics-18-00579-s001.zip › Supplementary Figures.pdf]

Pharmacokinetics of Monoclonal Antibodies in Pediatrics: Model-Based Investigation on Allometric Scaling Exponents

Elvis K. Danso [1]; Yuan Xiong [2*]; Mahesh N. Samtani [2]; Zhenhua Xu [1]

[1] Department of Clinical Pharmacology and Pharmacometrics, Johnson & Johnson Innovative Medicine, Spring House, PA 19002, USA
[2] Department of Clinical Pharmacology and Pharmacometrics, Johnson & Johnson Innovative Medicine, Raritan NJ 08869, USA

* Corresponding author: Yuan Xiong, yxiong6@its.jnj.com ; Tel: +1 215-628-5282

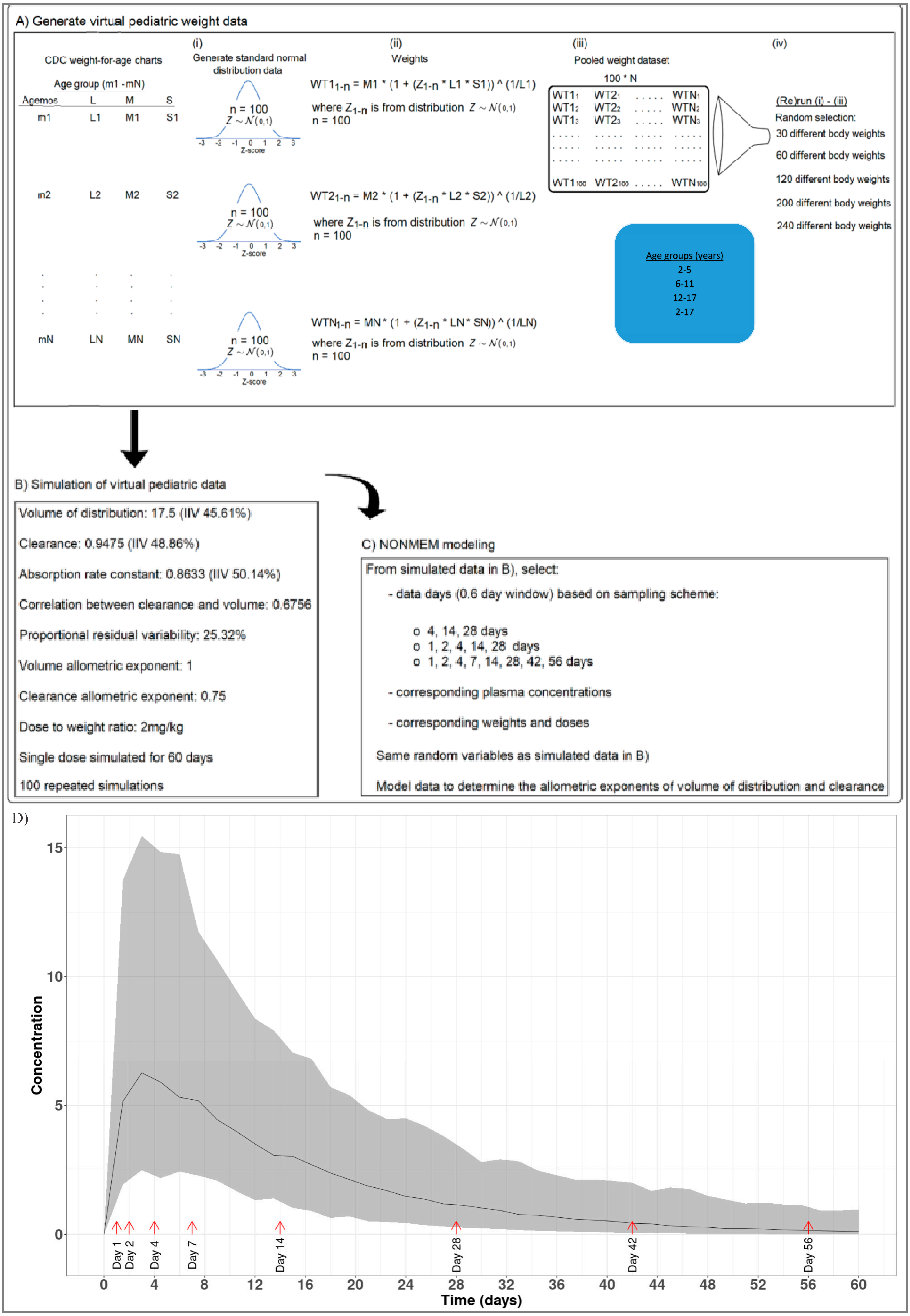

Supplementary Fig S1

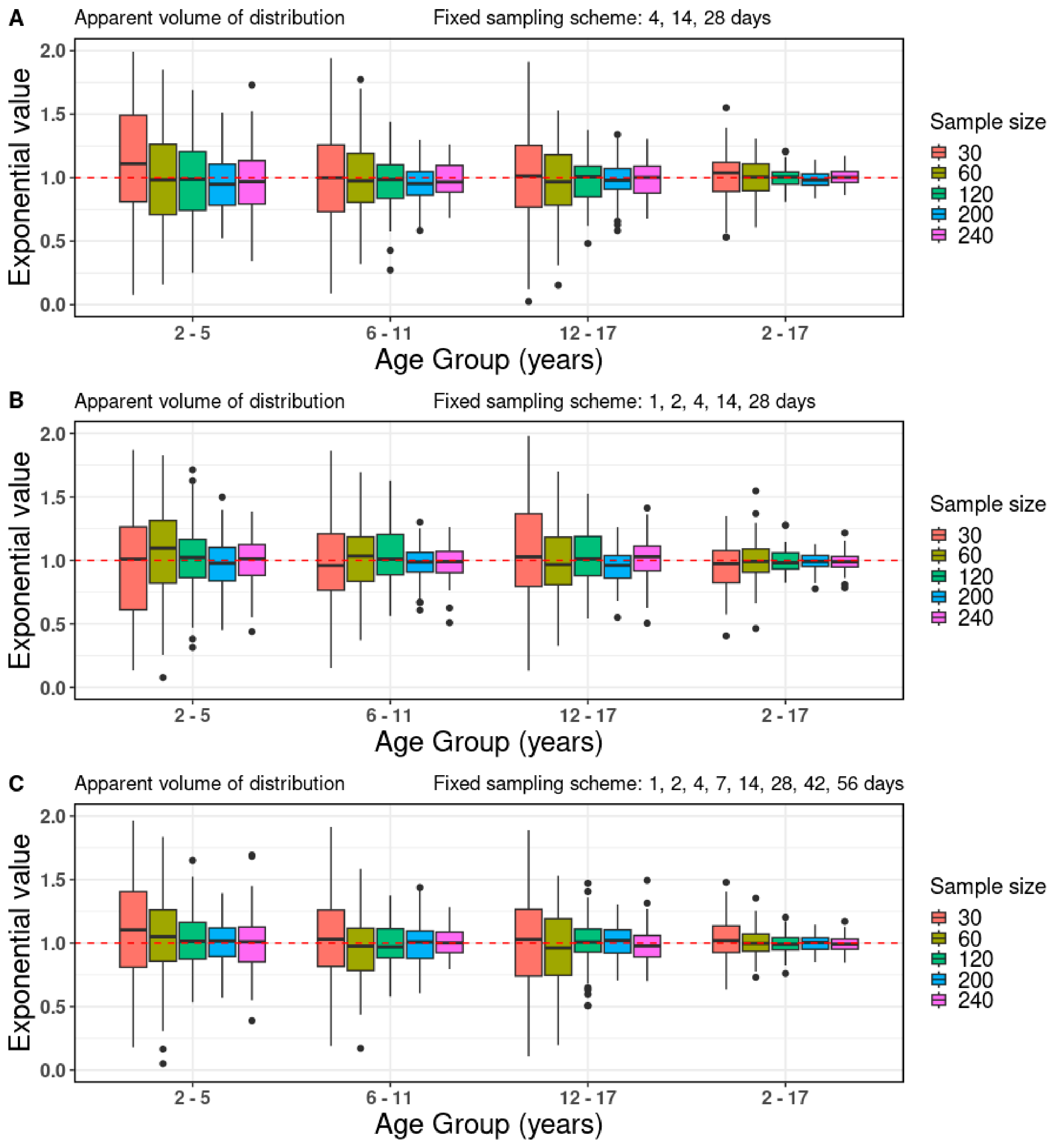

Supplementary Fig S2

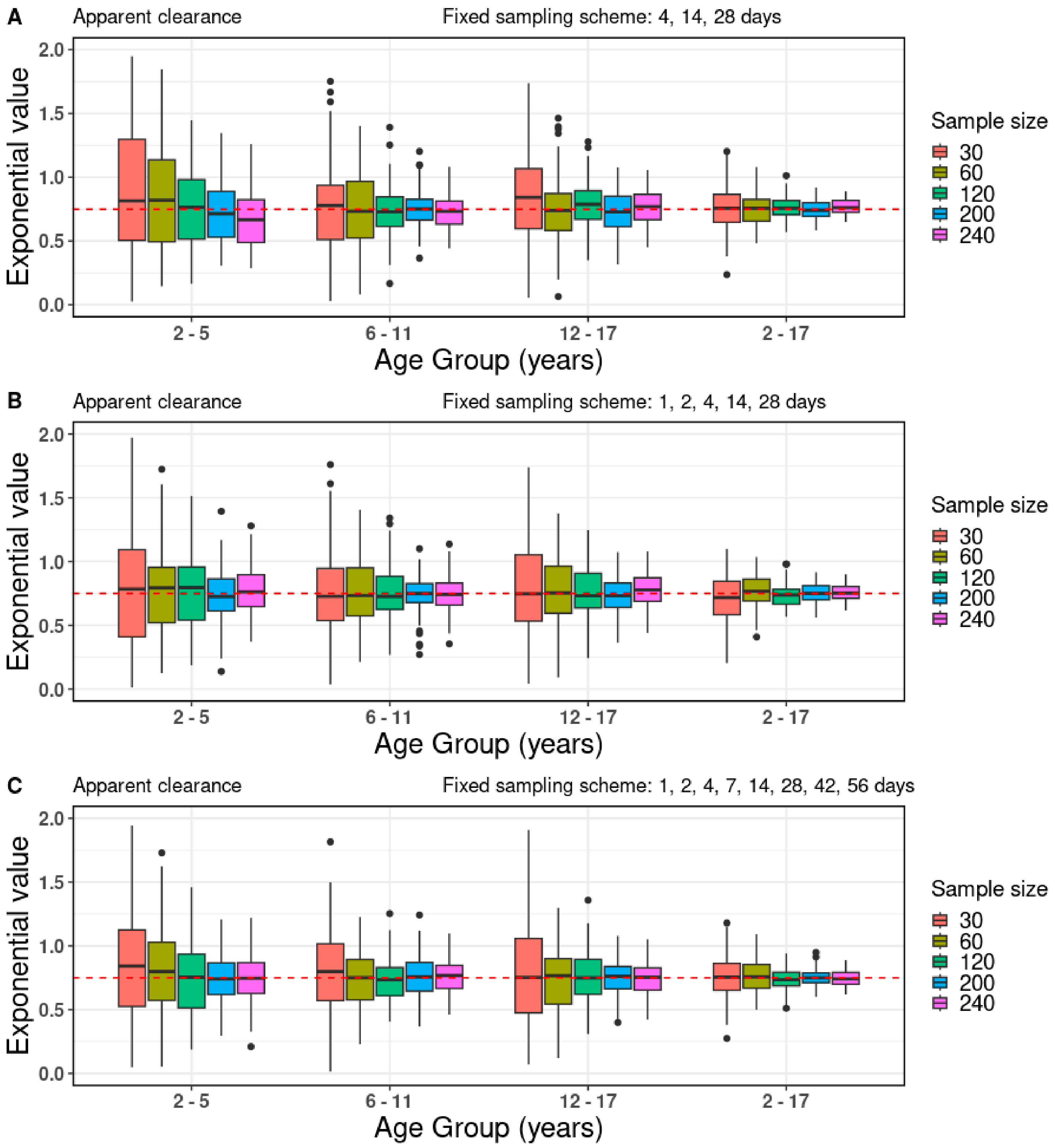

Supplementary Fig S3

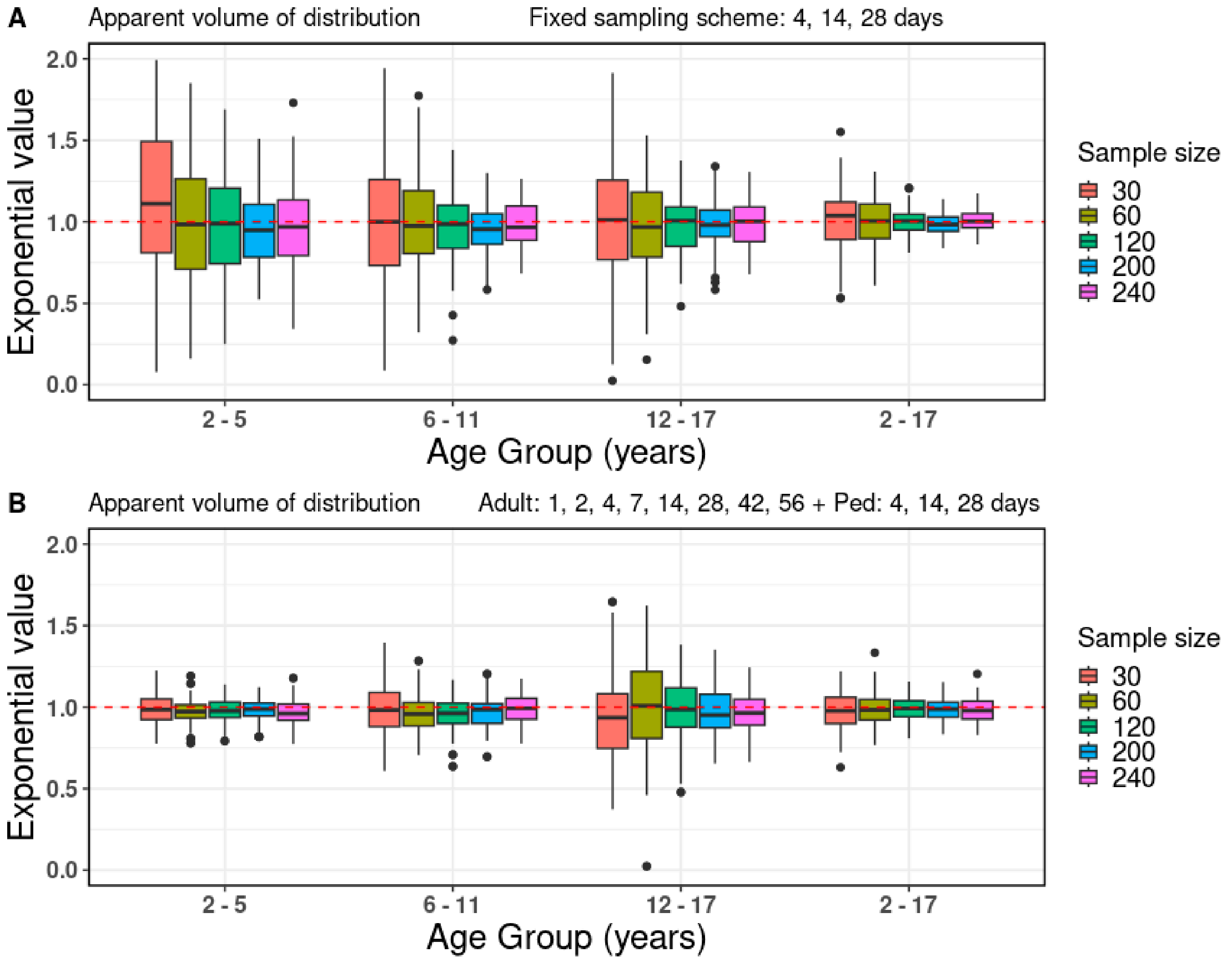

Supplementary Fig S4

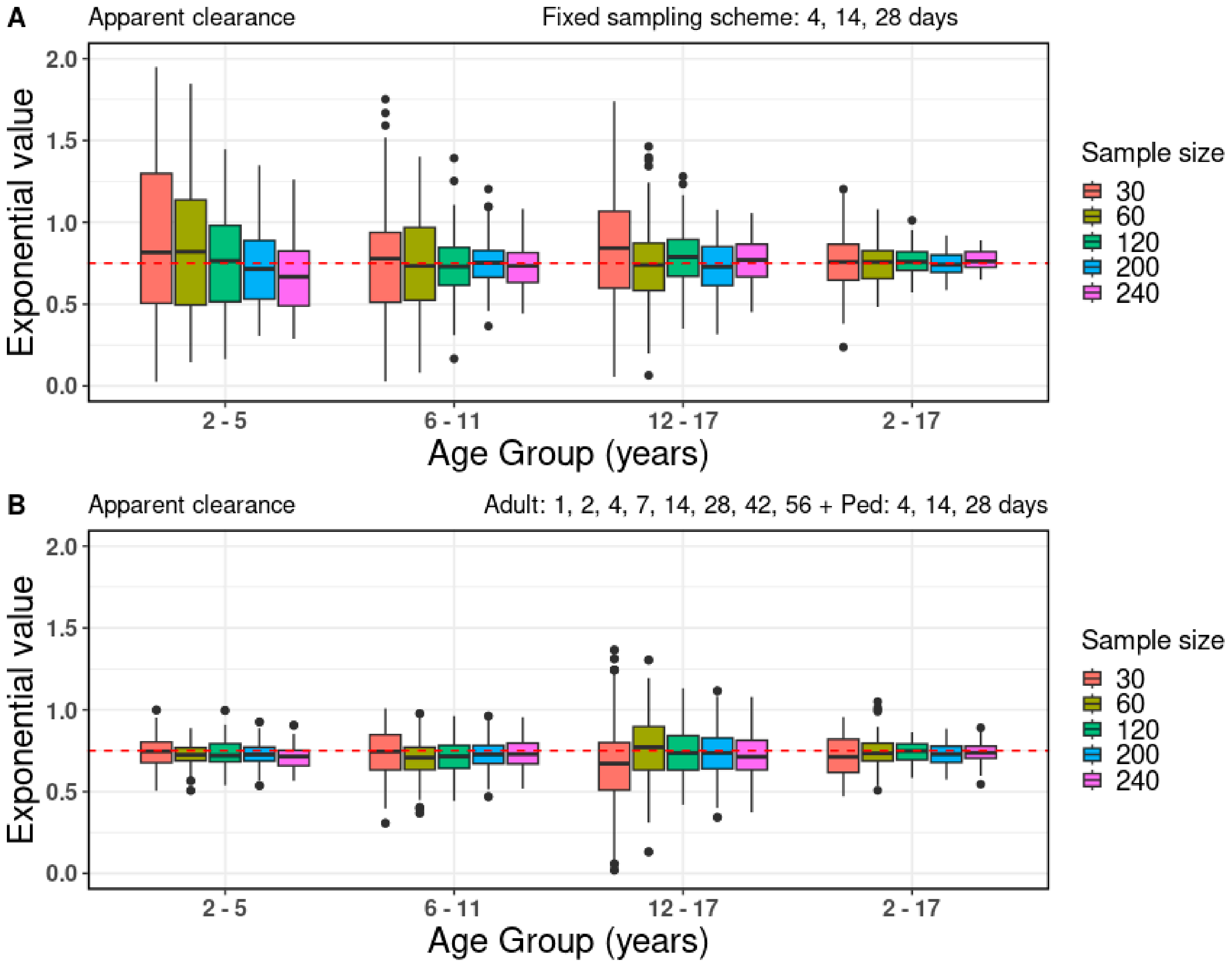

Supplementary Fig S5

Supplementary Figures

Supplementary Fig S1 Schematic of how pediatric data was generated, simulated, and modeled to determine allometric exponents. (A) Using data from CDC weight-for-age charts, one hundred weights were generated for each age (in month) record with a standard normal distribution ($weight = M(1 + ZLS)^{1/L}$). M represents median; Z represents Z score values generated from the standard normal distribution with 100 datapoints; L is power in the Box-Cox transformation; S is generalized coefficient of variation. All the individually generated one hundred weights for each age bracket were pooled together from which samples were randomly selected for 30, 60, 120, 200 and 240 different body weights. (B) Using RxODE, the generated pediatric data were simulated with the parameters provided (C) Using NONMEM, simulated data obtained from different sampling schemes and design factors were modeled to estimate the allometric exponents.

Supplementary Fig S2 Box-plot data of allometric exponent values of apparent volume of distribution for the virtual pediatric population for different age groups (2–5, 6–11, 12–17 and 2–17 y.o) and sample sizes of 30, 60, 120, 200 and 240, sampled on days (A) 4, 14, and 28 (B) 1, 2, 4, 14, and 28, and (C) 1, 2, 4, 7, 14, 28, 42, and 56. In the box plots, the central line represents the median, the box denotes the interquartile range (25th–75th percentiles), whiskers extend to the most extreme non-outlier values (within 1.5× interquartile range), and individual points indicate outliers. Red dotted lines indicate "true" value of allometric exponent of apparent volume of distribution of 1.0.

Supplementary Fig S3 Box-plot data of allometric exponent values of apparent clearance for the virtual pediatric population for different age groups (2–5, 6–11, 12–17 and 2–17 y.o) and sample sizes of 30, 60, 120, 200 and 240, sampled on days (A) 4, 14, and 28 (B) 1, 2, 4,14, and 28, and (C) 1, 2, 4, 7, 14, 28, 42, and 56. In the box plots, the central line represents the median, the box

denotes the interquartile range (25th–75th percentiles), whiskers extend to the most extreme non-outlier values (within 1.5× interquartile range), and individual points indicate outliers. Red dotted lines indicate “true” value of allometric exponent of apparent clearance of 0.75.

Supplementary Fig S4 Combined intensively sampled adult data (Phase 1 trial) and sparsely sample pediatric data (Phase 3 trial) to mimic clinical trial scenario. Box-plot data of allometric exponent values of apparent volume of distribution for the virtual pediatric population for different age groups (2–5, 6–11, 12–17 and 2–17 y.o) and sample sizes of 30, 60, 120, 200 and 240, sampled on days (A) 4, 14, and 28 for the pediatric data and (B) 1, 2, 4, 7, 14, 28, 42, and 56 for adult data combined with 4, 14, and 28 for pediatric data. In the box plots, the central line represents the median, the box denotes the interquartile range (25th–75th percentiles), whiskers extend to the most extreme non-outlier values (within 1.5× interquartile range), and individual points indicate outliers. Red dotted lines indicate “true” value of allometric exponent of apparent volume of distribution of 1.0. Peak concentration is achieved around day 4.

Supplementary Fig S5 Combined intensively sampled adult data (Phase 1 trial) and sparsely sample pediatric data (Phase 3 trial) to mimic clinical trial scenario. Box-plot data of allometric exponent values of apparent clearance for the virtual pediatric population for different age groups (2–5, 6–11, 12–17 and 2–17 y.o) and sample sizes of 30, 60, 120, 200 and 240, sampled on days (A) 4, 14 and 28 for the pediatric data and (B) 1, 2, 4, 7, 14, 28, 42, and 56 for adult data combined with 4, 14 and 28 for pediatric data. In the box plots, the central line represents the median, the box denotes the interquartile range (25th–75th percentiles), whiskers extend to the most extreme non-outlier values (within 1.5× interquartile range), and individual points indicate outliers. Red dotted lines indicate “true” value of allometric exponent of apparent clearance of 0.75. Peak concentration is achieved around day 4.
