# Supplementary material for: Pharmacokinetics of Monoclonal Antibodies in Pediatrics: Model-Based Investigation on Allometric Scaling Exponents
Source: Pharmaceutics. 2026 May 7;18(5):579. doi: 10.3390/pharmaceutics18050579 (PMC13210262; doi:10.3390/pharmaceutics18050579)
Supplement: Supplementary file 1 [file pharmaceutics-18-00579-s001.zip › Supplementary Tables.pdf]

Pharmacokinetics of Monoclonal Antibodies in Pediatrics: Model-Based Investigation on Allometric Scaling Exponents

Elvis K. Danso [1]; Yuan Xiong [2*]; Mahesh N. Samtani [2]; Zhenhua Xu [1]

[1] Department of Clinical Pharmacology and Pharmacometrics, Johnson & Johnson Innovative Medicine, Spring House, PA 19002, USA
[2] Department of Clinical Pharmacology and Pharmacometrics, Johnson & Johnson Innovative Medicine, Raritan NJ 08869, USA

* Corresponding author: Yuan Xiong, yxiong6@its.jnj.com ; Tel: +1 215-628-5282

Supplementary Table S1: Statistics of allometric exponent values of apparent volume of distribution for the virtual pediatric population for different age groups (2–5, 6–11, 12–17 and 2–17 y.o) and sample sizes of 30, 60, 120, 200 and 240, sampled on days (A) 4, 14, and 28 (B) 1, 2, 4, 14, and 28, and (C) 1, 2, 4, 7, 14, 28, 42, and 56.

		**2-5 years old**					**6-11 years old**				
	Sample size	30	60	120	200	240	30	60	120	200	240
Ped: 4, 14, 28	Median (25th-75th)	1.13 (0.83 - 1.57)	0.98 (0.71 - 1.26)	0.99 (0.74 - 1.21)	0.95 (0.78 - 1.11)	0.97 (0.79 - 1.13)	1 (0.74 - 1.28)	0.97 (0.81 - 1.19)	0.99 (0.84 - 1.1)	0.95 (0.86 - 1.05)	0.97 (0.89 - 1.1)
	Mean (SD)	1.19 (0.57)	1.01 (0.4)	0.98 (0.31)	0.95 (0.23)	0.97 (0.25)	1.01 (0.43)	0.98 (0.31)	0.98 (0.21)	0.96 (0.15)	0.97 (0.14)
Ped: 1, 2, 4, 14, 28	Median (25th-75th)	1.07 (0.66 - 1.34)	1.1 (0.82 - 1.31)	1.02 (0.86 - 1.17)	0.98 (0.84 - 1.1)	1.01 (0.88 - 1.12)	0.97 (0.77 - 1.21)	1.03 (0.83 - 1.19)	1.01 (0.89 - 1.2)	0.99 (0.91 - 1.06)	0.99 (0.9 - 1.07)
	Mean (SD)	1.08 (0.55)	1.06 (0.39)	1.01 (0.28)	0.98 (0.2)	1 (0.18)	1 (0.36)	1.02 (0.27)	1.02 (0.22)	0.98 (0.14)	0.99 (0.13)
Ped: 1, 2, 4, 7, 14, 28, 42, 56	Median (25th-75th)	1.11 (0.83 - 1.43)	1.05 (0.86 - 1.26)	1.01 (0.87 - 1.16)	1.02 (0.89 - 1.12)	1.01 (0.85 - 1.13)	1.03 (0.82 - 1.26)	0.98 (0.78 - 1.12)	0.97 (0.88 - 1.11)	1.01 (0.88 - 1.1)	1 (0.92 - 1.09)
	Mean (SD)	1.15 (0.48)	1.04 (0.39)	1.01 (0.24)	1.01 (0.17)	1 (0.22)	1.03 (0.35)	0.96 (0.25)	0.99 (0.16)	1 (0.17)	1.01 (0.11)

		**12-17 years old**					**2-17 years old**				
	Sample size	30	60	120	200	240	30	60	120	200	240
Ped: 4, 14, 28	Median (25th-75th)	1.01 (0.77 - 1.25)	0.97 (0.78 - 1.18)	1.01 (0.85 - 1.09)	0.98 (0.91 - 1.07)	1 (0.88 - 1.09)	1.04 (0.89 - 1.12)	1 (0.9 - 1.11)	1 (0.95 - 1.05)	0.98 (0.94 - 1.03)	1 (0.96 - 1.05)
	Mean (SD)	1.01 (0.38)	0.98 (0.3)	0.97 (0.18)	0.99 (0.16)	1 (0.15)	1.01 (0.19)	1 (0.15)	1 (0.09)	0.99 (0.07)	1 (0.06)
Ped: 1, 2, 4, 14, 28	Median (25th-75th)	1.03 (0.79 - 1.37)	0.97 (0.81 - 1.18)	1.01 (0.88 - 1.19)	0.96 (0.86 - 1.04)	1.03 (0.92 - 1.11)	0.97 (0.82 - 1.08)	0.99 (0.91 - 1.09)	0.98 (0.93 - 1.06)	0.99 (0.95 - 1.04)	0.99 (0.95 - 1.03)
	Mean (SD)	1.05 (0.42)	0.98 (0.29)	1.03 (0.21)	0.96 (0.14)	1.01 (0.15)	0.95 (0.18)	0.99 (0.15)	0.99 (0.09)	0.99 (0.07)	0.99 (0.07)
Ped: 1, 2, 4, 7, 14, 28, 42, 56	Median (25th-75th)	1.06 (0.74 - 1.28)	0.96 (0.75 - 1.19)	1.01 (0.93 - 1.11)	1.02 (0.92 - 1.1)	0.98 (0.89 - 1.06)	1.02 (0.93 - 1.13)	1 (0.94 - 1.07)	0.99 (0.95 - 1.04)	1 (0.95 - 1.04)	0.99 (0.95 - 1.03)
	Mean (SD)	1.05 (0.41)	0.97 (0.27)	1 (0.18)	1.01 (0.13)	0.98 (0.13)	1.03 (0.17)	1 (0.12)	0.99 (0.08)	1 (0.06)	0.99 (0.06)

Supplementary Table S2: Statistics of allometric exponent values of apparent clearance for the virtual pediatric population for different age groups (2–5, 6–11, 12–17 and 2–17 y.o) and sample sizes of 30, 60, 120, 200 and 240, sampled on days (A) 4, 14, and 28 (B) 1, 2, 4,14, and 28, and (C) 1, 2, 4, 7, 14, 28, 42, and 56.

		**2-5 years old**					**6-11 years old**				
	Sample size	30	60	120	200	240	30	60	120	200	240
Ped: 4, 14, 28	Median (25th-75th)	0.82 (0.51 - 1.3)	0.82 (0.49 - 1.14)	0.76 (0.51 - 0.98)	0.72 (0.53 - 0.89)	0.67 (0.49 - 0.82)	0.78 (0.51 - 0.94)	0.73 (0.52 - 0.97)	0.73 (0.62 - 0.85)	0.75 (0.66 - 0.83)	0.73 (0.63 - 0.81)
	Mean (SD)	0.91 (0.51)	0.84 (0.39)	0.75 (0.31)	0.72 (0.23)	0.7 (0.25)	0.76 (0.35)	0.74 (0.29)	0.75 (0.19)	0.76 (0.16)	0.73 (0.13)
Ped: 1, 2, 4, 14, 28	Median (25th-75th)	0.78 (0.41 - 1.09)	0.8 (0.52 - 0.96)	0.8 (0.54 - 0.96)	0.73 (0.61 - 0.86)	0.76 (0.65 - 0.9)	0.73 (0.54 - 0.95)	0.73 (0.57 - 0.95)	0.73 (0.62 - 0.88)	0.75 (0.68 - 0.83)	0.74 (0.66 - 0.83)
	Mean (SD)	0.8 (0.45)	0.78 (0.34)	0.76 (0.29)	0.74 (0.21)	0.78 (0.18)	0.76 (0.37)	0.77 (0.26)	0.76 (0.21)	0.74 (0.15)	0.75 (0.14)
Ped: 1, 2, 4, 7, 14, 28, 42, 56	Median (25th-75th)	0.85 (0.53 - 1.13)	0.8 (0.57 - 1.03)	0.75 (0.51 - 0.94)	0.74 (0.62 - 0.87)	0.75 (0.63 - 0.87)	0.8 (0.57 - 1.02)	0.75 (0.58 - 0.89)	0.73 (0.61 - 0.83)	0.76 (0.65 - 0.87)	0.77 (0.67 - 0.85)
	Mean (SD)	0.87 (0.49)	0.81 (0.33)	0.74 (0.26)	0.74 (0.19)	0.75 (0.2)	0.79 (0.33)	0.74 (0.24)	0.73 (0.17)	0.76 (0.17)	0.76 (0.13)

		**12-17 years old**					**2-17 years old**				
	Sample size	30	60	120	200	240	30	60	120	200	240
Ped: 4, 14, 28	Median (25th-75th)	0.84 (0.6 - 1.07)	0.74 (0.58 - 0.87)	0.79 (0.67 - 0.9)	0.73 (0.61 - 0.85)	0.77 (0.67 - 0.87)	0.76 (0.65 - 0.87)	0.76 (0.66 - 0.83)	0.76 (0.71 - 0.82)	0.74 (0.69 - 0.8)	0.76 (0.72 - 0.82)
	Mean (SD)	0.84 (0.36)	0.74 (0.27)	0.79 (0.18)	0.73 (0.17)	0.77 (0.13)	0.75 (0.17)	0.75 (0.12)	0.76 (0.08)	0.74 (0.07)	0.77 (0.06)
Ped: 1, 2, 4, 14, 28	Median (25th-75th)	0.75 (0.53 - 1.05)	0.75 (0.59 - 0.96)	0.73 (0.64 - 0.91)	0.73 (0.64 - 0.83)	0.78 (0.69 - 0.87)	0.72 (0.58 - 0.85)	0.77 (0.69 - 0.86)	0.74 (0.67 - 0.78)	0.75 (0.7 - 0.81)	0.75 (0.71 - 0.8)
	Mean (SD)	0.8 (0.38)	0.77 (0.26)	0.76 (0.18)	0.73 (0.15)	0.78 (0.13)	0.72 (0.19)	0.76 (0.13)	0.74 (0.09)	0.75 (0.07)	0.76 (0.07)
Ped: 1, 2, 4, 7, 14, 28, 42, 56	Median (25th-75th)	0.76 (0.48 - 1.07)	0.77 (0.54 - 0.9)	0.75 (0.62 - 0.89)	0.76 (0.66 - 0.84)	0.75 (0.65 - 0.83)	0.75 (0.65 - 0.86)	0.76 (0.67 - 0.85)	0.73 (0.69 - 0.79)	0.75 (0.71 - 0.79)	0.74 (0.7 - 0.79)
	Mean (SD)	0.78 (0.4)	0.72 (0.26)	0.78 (0.19)	0.75 (0.13)	0.74 (0.14)	0.75 (0.18)	0.77 (0.13)	0.74 (0.09)	0.75 (0.07)	0.74 (0.06)

Supplementary Table S3: Statistics of allometric exponent values of apparent volume of distribution for the virtual pediatric population for different age groups (2–5, 6–11, 12–17 and 2–17 y.o) and sample sizes of 30, 60, 120, 200 and 240, sampled on days (A) 4, 14, and 28 for the pediatric data and (B) 1, 2, 4, 7, 14, 28, 42, and 56 for adult data combined with 4, 14, and 28 for pediatric data.

		**2-5 years old**					**6-11 years old**				
	Sample size	30	60	120	200	240	30	60	120	200	240
Ped: 4, 14, 28	Median (25th-75th)	1.13 (0.83 - 1.57)	0.98 (0.71 - 1.26)	0.99 (0.74 - 1.21)	0.95 (0.78 - 1.11)	0.97 (0.79 - 1.13)	1 (0.74 - 1.28)	0.97 (0.81 - 1.19)	0.99 (0.84 - 1.1)	0.95 (0.86 - 1.05)	0.97 (0.89 - 1.1)
	Mean (SD)	1.19 (0.57)	1.01 (0.4)	0.98 (0.31)	0.95 (0.23)	0.97 (0.25)	1.01 (0.43)	0.98 (0.31)	0.98 (0.21)	0.96 (0.15)	0.97 (0.14)
Adult: 1, 2, 4, 7, 14, 28, 42, 56, Ped: 4, 14, 28	Median (25th-75th)	0.98 (0.92 - 1.05)	0.97 (0.93 - 1.01)	0.98 (0.94 - 1.03)	0.99 (0.95 - 1.03)	0.96 (0.92 - 1.02)	0.98 (0.88 - 1.09)	0.96 (0.88 - 1.03)	0.96 (0.9 - 1.02)	0.98 (0.9 - 1.02)	0.99 (0.93 - 1.05)
	Mean (SD)	0.99 (0.09)	0.97 (0.07)	0.98 (0.07)	0.98 (0.07)	0.96 (0.08)	0.98 (0.15)	0.96 (0.12)	0.96 (0.1)	0.97 (0.09)	0.99 (0.09)

		**12-17 years old**					**2-17 years old**				
	Sample size	30	60	120	200	240	30	60	120	200	240
Ped: 4, 14, 28	Median (25th-75th)	1.01 (0.77 - 1.25)	0.97 (0.78 - 1.18)	1.01 (0.85 - 1.09)	0.98 (0.91 - 1.07)	1 (0.88 - 1.09)	1.04 (0.89 - 1.12)	1 (0.9 - 1.11)	1 (0.95 - 1.05)	0.98 (0.94 - 1.03)	1 (0.96 - 1.05)
	Mean (SD)	1.01 (0.38)	0.98 (0.3)	0.97 (0.18)	0.99 (0.16)	1 (0.15)	1.01 (0.19)	1 (0.15)	1 (0.09)	0.99 (0.07)	1 (0.06)
Adult: 1, 2, 4, 7, 14, 28, 42, 56, Ped: 4, 14, 28	Median (25th-75th)	0.94 (0.75 - 1.08)	1.01 (0.81 - 1.22)	0.99 (0.88 - 1.12)	0.95 (0.87 - 1.08)	0.96 (0.89 - 1.05)	0.98 (0.9 - 1.06)	0.98 (0.92 - 1.05)	0.99 (0.94 - 1.04)	0.99 (0.94 - 1.03)	0.98 (0.93 - 1.04)
	Mean (SD)	0.94 (0.26)	1 (0.27)	0.99 (0.17)	0.98 (0.14)	0.97 (0.13)	0.97 (0.12)	0.98 (0.1)	0.99 (0.07)	0.98 (0.06)	0.98 (0.07)

Supplementary Table S4: Statistics of allometric exponent values of apparent clearance for the virtual pediatric population for different age groups (2–5, 6–11, 12–17 and 2–17 y.o) and sample sizes of 30, 60, 120, 200 and 240, sampled on days (A) 4, 14 and 28 for the pediatric data and (B) 1, 2, 4, 7, 14, 28, 42, and 56 for adult data combined with 4, 14 and 28 for pediatric data.

		**2-5 years old**					**6-11 years old**				
	Sample size	30	60	120	200	240	30	60	120	200	240
Ped: 4, 14, 28	Median (25th-75th)	0.82 (0.51 - 1.3)	0.82 (0.49 - 1.14)	0.76 (0.51 - 0.98)	0.72 (0.53 - 0.89)	0.67 (0.49 - 0.82)	0.78 (0.51 - 0.94)	0.73 (0.52 - 0.97)	0.73 (0.62 - 0.85)	0.75 (0.66 - 0.83)	0.73 (0.63 - 0.81)
	Mean (SD)	0.91 (0.51)	0.84 (0.39)	0.75 (0.31)	0.72 (0.23)	0.7 (0.25)	0.76 (0.35)	0.74 (0.29)	0.75 (0.19)	0.76 (0.16)	0.73 (0.13)
Adult: 1, 2, 4, 7, 14, 28, 42, 56, Ped: 4, 14, 28	Median (25th-75th)	0.75 (0.68 - 0.8)	0.72 (0.69 - 0.77)	0.72 (0.68 - 0.79)	0.73 (0.69 - 0.77)	0.71 (0.66 - 0.75)	0.74 (0.63 - 0.85)	0.71 (0.63 - 0.77)	0.72 (0.64 - 0.78)	0.73 (0.67 - 0.78)	0.73 (0.67 - 0.8)
	Mean (SD)	0.74 (0.09)	0.73 (0.07)	0.73 (0.09)	0.73 (0.07)	0.71 (0.07)	0.73 (0.16)	0.7 (0.12)	0.71 (0.11)	0.72 (0.09)	0.73 (0.09)

		**12-17 years old**					**2-17 years old**				
	Sample size	30	60	120	200	240	30	60	120	200	240
Ped: 4, 14, 28	Median (25th-75th)	0.84 (0.6 - 1.07)	0.74 (0.58 - 0.87)	0.79 (0.67 - 0.9)	0.73 (0.61 - 0.85)	0.77 (0.67 - 0.87)	0.76 (0.65 - 0.87)	0.76 (0.66 - 0.83)	0.76 (0.71 - 0.82)	0.74 (0.69 - 0.8)	0.76 (0.72 - 0.82)
	Mean (SD)	0.84 (0.36)	0.74 (0.27)	0.79 (0.18)	0.73 (0.17)	0.77 (0.13)	0.75 (0.17)	0.75 (0.12)	0.76 (0.08)	0.74 (0.07)	0.77 (0.06)
Adult: 1, 2, 4, 7, 14, 28, 42, 56, Ped: 4, 14, 28	Median (25th-75th)	0.67 (0.51 - 0.8)	0.77 (0.63 - 0.9)	0.74 (0.63 - 0.84)	0.74 (0.64 - 0.83)	0.71 (0.63 - 0.81)	0.71 (0.62 - 0.82)	0.73 (0.69 - 0.8)	0.75 (0.69 - 0.79)	0.73 (0.68 - 0.78)	0.74 (0.7 - 0.78)
	Mean (SD)	0.67 (0.29)	0.76 (0.21)	0.74 (0.16)	0.74 (0.15)	0.72 (0.14)	0.71 (0.12)	0.74 (0.1)	0.74 (0.07)	0.73 (0.07)	0.74 (0.06)
